# Cognitive and Typing Outcomes Measured Simultaneously with Slow Treadmill Walking or Sitting: Implications for Treadmill Desks

**DOI:** 10.1371/journal.pone.0121309

**Published:** 2015-04-15

**Authors:** Michael J. Larson, James D. LeCheminant, Kyle Hill, Kaylie Carbine, Travis Masterson, Ed Christenson

**Affiliations:** 1 Department of Psychology and Neuroscience Center, Brigham Young University, Provo, Utah, United States of America; 2 Department of Exercise Sciences, Brigham Young University, Provo, Utah, United States of America; University of Bath, UNITED KINGDOM

## Abstract

**Purpose:**

This study compared cognitive (attention, learning, and memory) and typing outcomes during slow treadmill walking or sitting. Seventy-five healthy individuals were randomly assigned to a treadmill walking group (n=37; 23 female) or sitting group (n=38; 17 female).

**Methods:**

The treadmill walking group completed a series of tests while walking at 1.5 mph. The sitting group performed the same tests while sitting at a standard desk. Tests performed by both groups included: the Rey Auditory Verbal Learning Test and a modified version of the Paced Auditory Serial Attention Test. In addition, typing performance was evaluated.

**Results:**

Participants in the treadmill walking group performed worse on the Rey Auditory Verbal Learning Test for total learning than the sitting group; the main effect was significant (*F*(1,73)=4.75, *p*=0.03, *η*_*p*_^*2*^=0.06); however, short- and long-delay recall performance did not differ between groups (p>0.05). For the Paced Auditory Serial Attention Test, total number of correct responses was lower in the treadmill walking group relative to the sitting group; the main effect was significant (*F*(1,73)=4.97, *p*=0.03, *η*_*p*_^*2*^=0.06). The performance of both groups followed the same learning slope (Group x Trial interactions were not significant) for the Rey Auditory Verbal Learning Test and Paced Auditory Serial Attention Test. Individuals in the treadmill walking group performed significantly worse for all measures of typing (*p*<0.05).

**Conclusion:**

Walking on a treadmill desk may result in a modest difference in total learning and typing outcomes relative to sitting, but those declines may not outweigh the benefit of the physical activity gains from walking on a treadmill.

## Introduction

Current physical activity and health guidelines suggest that adults engage in 150 minutes of moderate-intensity or 75 minutes of vigorous-intensity activity per week [1]. Compared to inactive adults, physically-active adults tend to have reduced all-cause mortality, and decreased risk of several chronic diseases and related risk factors [2–6]. Independent of physical activity level, prolonged sedentary time (activities 1.0–1.5 METs), such as sitting and television viewing, may also adversely affect some health outcomes [7–10]. Specifically, sedentary individuals may be at higher risk for obesity [11–14]. Obesity is a prevalent public health concern that affects approximately 35% of adults in the U.S. and increases the risk of high blood pressure, chronic diseases, cancer, and mortality [11,15]. Consequently, there is a pressing need to develop interventions that promote physical health by decreasing sedentary behavior.

For many adults, sedentariness is particularly prevalent at the workplace. Given emerging evidence that increasing physical activity may positively influence some aspects of productivity [16] and reduce cardiovascular disease, type 2 diabetes, and obesity [17], worksites have received increasing attention as a venue to increase physical activity and reduce sedentary time [18]. Recent studies have implemented novel approaches to reduce sedentary time at the workplace, such as sit-stand desks (desks that can be transformed to allow for either sitting or standing), treadmills desks (desks equipped with a treadmill for slow walking during workplace activities), and stepping devices [19–23].

Treadmill desks, in particular, have received increased attention in the literature. Several studies indicate that incorporation of a treadmill desk in the workplace significantly increases physical activity, increases energy expenditure, and/or reduces sedentary time [19,24,25]. Treadmill workstations may be particularly advantageous as they increase energy expenditure to a greater extent than other alternative workstations, such as sit-stand desks or stability balls [26]. This is positive; however, research examining the simultaneous effect of slow treadmill walking and cognitive performance (math, reading, attention, processing speed, memory, etc.) and/or work-related tasks (e.g., typing) is sparse. These outcomes are important with consideration to productivity. Presently, the literature is limited by too few studies (particularly for cognitive outcomes), differing measurements, and mixed results [24,26–30]. For example, studies have suggested that math and reading performance [27], along with computer and typing tasks [23,30] were worse while using a treadmill workstation compared to sitting at a desk. On the other hand, others have reported that cognitive performance or typing performance were not different while treadmill walking versus sitting [28,29]. Thus, the effect of using a treadmill desk workstation on important outcomes, related to work performance, is presently unclear. This was recently confirmed in a comprehensive review by Tudor-Locke and colleagues who noted, “large gaps in scientific understanding” relative to workstations that include treadmill desks [26].

Our knowledge of the influence of concurrent treadmill walking and cognitive performance is informed by studies of the effects of acute exercise on subsequent cognition. Meta-analyses suggest a small but positive effect of exercise on cognition during, immediately following, and long-term after a single exercise bout [31–33]. The effects of exercise on cognition are most pronounced at moderate-intensity exercise (compared to low- or high-intensity exercise) when looking at cognitive tasks that have a significant speed-related component, but effect sizes are near zero for low, moderate, and high-intensity exercise when looking at task-related accuracy [33]. Notably, many studies in this area utilize small samples, do not randomize groups to conditions, and use simple tasks such as a flanker or symbol-digit modalities test that are based on speed of response, but have a large negative skew where accuracy performance may be at ceiling levels [33].

The current study was specifically designed to examine the effects of treadmill walking at a speed consistent with workplace treadmill desk use, rather than examining the acute effects of different exercise speeds or intensities on subsequent cognitive performance. We also utilized tests derived from the psychometric tradition of clinical neuropsychology that look at errors and recall of information, rather than focusing on speed of response as the key outcome variable, and are applicable to individuals in the workplace setting who may use treadmill desks. We specifically chose tasks reliant on attention, working memory, learning, and memory functions as these are skills relied upon in the workplace. Tests are described in detail below and included the Paced Auditory Serial Addition Test (PASAT) and the Rey Auditory Verbal Learning Test (AVLT) [34–36]. The PASAT is standardized measure of working memory and attention that loads onto attention, general intelligence, and freedom from distraction factors [37]. Notably, the PASAT requires rapid processing and responding and is very sensitive to novelty and practice effects [38]. The AVLT is sensitive to difficulties in memory and learning [39] and requires attention processes during the initial learning trials as well as memory abilities to retain information over a delay [35]. Performance on the AVLT declines in workplace environments that are stressful or fatiguing, such as shift work and is associated with oxidative stress [40]. In summary, the current study compared cognitive (attention, working memory, learning, and memory) and typing outcomes during slow treadmill walking (1.5 mph using a treadmill desk) or sitting (using a standard desk). Based on the current literature, we hypothesized that those in the treadmill-walking group would perform moderately worse or not different than those in the sitting group in the domains of attention, learning, memory, and typing speed.

## Methods

The Institutional Review Board in the Office of Research and Creative Activities at Brigham Young University approved all study procedures and all participants provided written informed consent before participation. We utilized a randomized experimental design with two separate groups. Outcome variables (described below) included measures of attention/working memory and learning and memory, along with a measure of typing speed and accuracy. All participants were tested between the hours of 7am and 10am.

### Participants

Seventy-six healthy participants were enrolled in the study and randomly assigned to a sitting group or treadmill walking group. One participant initially assigned to the sitting group did not complete testing due to computer malfunction. Thus, final study enrollment included 75 participants—38 (17 female) assigned to the sitting group and 37 (23 female) assigned to the treadmill walking group. All participants were recruited from large undergraduate courses at the local institution. Participant characteristics are presented in Table 1.

**Table 1:** Demographic characteristics of sitting and treadmill walking participants.

	Sitting *n* = 38	Treadmill *n* = 37	*t or* χ^2^	*p* value
Sex (female), n (%)	17 (45%)	23 (62%)	2.29	0.13
Age (yrs)	20.71 (2.12)	20.84 (2.37)	0.25	0.81
Weight (kg)	67.08 (11.72)	70.22 (13.07)	1.09	0.28
Height (cm)	172.50 (9.71)	173.87 (8.71)	0.64	0.52
Body mass index (kg/m^2^)	22.44 (2.72)	23.16 (3.87)	0.94	0.35
VO_2_ peak (ml/kg/min)	57.08 (7.63)	58.53 (7.15)	0.84	0.41

Data include mean (SD).

Inclusion and exclusion criteria were assessed via participant self-report. Participants were between the ages of 18 and 35 years, attended at least some college, native English speakers, and able to type at least 25 words per minute. Exclusion criteria included the presence of any chronic or metabolic disease, diagnosis of or medication for a psychiatric disorder (e.g., major depressive disorder), inconsistent sleep patterns, alcohol use, nicotine use, or substance abuse within the past year, pregnancy or current lactation, physical injury/disability that limited jogging, or a neurological disorder such as traumatic brain injury, epilepsy, or attention-deficit/hyperactivity disorder.

### Study Procedures

Participants were instructed to arrive in a fasted state (i.e., no food or caloric beverage after 9pm the previous night), no caffeine or vigorous exercise for 24-hours prior to testing, and after at least 7 hours of sleep the previous night. Participants were asked to arrive in a fasted stated to control for potential differences in caloric intake and hunger levels that previous research suggests can influence cognitive performance [41]. These criteria were verbally confirmed upon arrival at the laboratory. Participants then completed consent procedures and were randomly assigned to a study group using a random number generator. Participants subsequently completed a demographic questionnaire; height was measured using a standard wall-mounted stadiometer (Seca, Chino, CA) and body weight was assessed using a digital scale (Tanita Corporation, Japan), accurate to the nearest hundredth pound.

Participants in the treadmill walking group received instruction for treadmill safety and a familiarization period of ~5 minutes of treadmill walking. Next, the participants (including those in the sitting group) received brief instruction of the testing procedures. Subsequently, each participant completed the experimental measures while either walking on the treadmill or sitting (measures described in detail below). A flat desk was positioned over the treadmill creating a usable treadmill desk-workstation. The desk portion was 48.5 in (123 cm) tall, 37 in (94 cm) wide, and 24 in (61 cm) deep; sufficient size for a computer monitor and keyboard needed for the cognitive tests performed in this study. Participants in the treadmill walking group completed all tasks while walking on a motor-driven treadmill (LifeSpan, Salt Lake City, UT), set to a speed of 1.5 mph and 0% grade. This speed is consistent with the present literature using treadmill speeds between 0.5 and 2.5 mph [26]. Participants in the sitting group utilized a standard sitting desk. Total time either walking or sitting was ~45 minutes.

All participants first completed the initial learning trials of the Rey Auditory Verbal Learning Test (AVLT), a test of learning and memory, to provide adequate time for the 30-minute delay required for this test. The remaining study measures, including the Paced Auditory Serial Attention Test (PASAT) and typing test, were completed in randomized order, with the recall trials of the AVLT completed after at least a 30-minute delay. Trained research assistants administered the AVLT and PASAT; participants provided oral responses to the test stimuli.

### Outcome Measures

#### Rey Auditory Verbal Learning Test (AVLT)

The AVLT measures learning and memory using two 15-item word lists [34]. The AVLT was administered in standardized fashion. Specifically, participants were initially presented with five learning trials. A distracter list was then presented followed by a short-delay free recall trial. After a 30-minute delay, participants were asked to recall as many words as possible. Learning was examined using the first five learning trials. Recall was examined using the number of words recalled after the initial distractor list (Short Delay Recall) and the number of words recalled after the 30-minute delay (Long Delay Recall). The AVLT has good test-retest reliability for time-frames as long as a year (up to 0.70) and correlates significantly with other measures of learning and memory (>0.50) [42].

#### Paced Auditory Serial Addition Test (PASAT)

Participants completed a modified version of the PASAT, a test that measures processing speed, attention, and working memory abilities. For this test, audiotaped numbers from 1 to 9 were presented at rates of 2.4 seconds (block 1), 2.0 seconds (block 2), 1.6 seconds (block 3), and 1.2 seconds (block 4). Fifty-one numbers (50 responses per block for a total of 200 possible responses) were presented for four separate trial blocks [36]. Participants were instructed to sum the first pair of numbers together, and then add the following number sequentially. For example, if the numbers presented were “3, 5” participants would sum 3 and 5 to verbally answer “8”; if the next number presented was 6, the participant would sum 6 and 5 to verbally answer “11”. We used the number correct instead of accuracy due to response omissions during performance that reflects a strategy commonly seen when individuals are trying to compensate for the speed of the task [43]. The PASAT has high internal consistency (approximately 0.90) and test-retest reliability (0.78 to 0.83) [42,44].

#### Typing Test

Typing speed was assessed using the TypingMaster Pro Version 6.3. Participants typed words presented on the screen for 5 minutes as quickly and accurately as possible. Three outcome variables were assessed: 1) typing gross words per minute (Gross WPM); 2) net typing words per minute (Net WPM), wherein the number of incorrect words were subtracted from the gross typing speed; and 3) percent typing accuracy (Accuracy) calculated by dividing the Net WPM by the Gross WPM.

#### Cardiovascular Fitness (estimated VO_2_ peak)

We used a submaximal jogging protocol and equation validated by George et al. [45] to predict maximal aerobic capacity. Submaximal tests are less strenuous and typically take less time than a maximal aerobic capacity test; thus, are preferred by many research participants. For this test, each participant achieved a submaximal jogging pace between 4.3–7.5 mph (≤6.5 for females and ≤7.5 for males) for 3 continuous minutes of steady state heart rate. Steady state heart rate levels were valid only if less than 180 beats per minute. Cross-validation of this test compared to a maximal test showed a correlation of *r* = 0.88, SEE = 3.1 ml/kg/min [45]. Notably, the fitness task occurred after all other study measures were completed so as not to confound the cognitive performance results.

### Statistical Analysis

Descriptive statistics for age, body weight, BMI, VO_2_ peak, and typing speed were reported as means and standard deviations and compared between groups using independent-samples *t*-tests. The primary outcomes of interest for this study were the between-group differences for total learning (AVLT) and total number of correct responses on the PASAT. We also examined group differences by trial (time). A repeated-measures analysis of variance (ANOVAs) was used to determine main effects of group or trial, as well as the presence of a Group x Trial interaction, for the AVLT and PASAT. Further, consistent with previous research [46,47], we conducted two separate repeated measures ANOVAs for the AVLT. The first focused on learning trials and included the first five AVLT list presentations. The second focused on recall and included the short- and long-delay recall trials. The ANOVA for the PASAT included the four trial blocks. Partial-eta^2^ ($\eta_{p}^{2}$) is presented for ANOVA effect sizes. Standardized *z*-scores as a function of group were calculated by subtracting each individual participant score from age-corrected normative scores and dividing by the norm group standard deviation. Normative scores for the AVLT were obtained from the meta-norms for the AVLT compiled by Schmidt [48]. Normative scores for the PASAT were obtained from Roman et al.’s extended norms for the PASAT [49]. Cohen’s-*d* is presented for between-group effect sizes for typing outcomes; *d-*values of 0.20 indicate small effects, 0.50 indicate medium effects, and 0.80 indicate large effects [50].

## Results

Participants in the treadmill and sitting groups did not significantly differ in demographic characteristics, including male-to-female ratio, age, weight, height, BMI, or VO_2_ peak (Table 1).

Cognitive outcome measures are presented in Table 2. Participants in the treadmill walking group performed worse on the AVLT for total learning than the sitting group; the main effect was significant, *F*(1,73) = 4.75, *p* = 0.03, $\eta_{p}^{2}$= 0.06. A 2-Group x 5-Trial (five learning trials) repeated measures ANOVA revealed a significant main effect of trial, *F*(4,292) = 294.59, *p*<0.001, $\eta_{p}^{2}$= 0.80, both groups learned more words with each subsequent trial. The Group x Trial interaction, however, was not significant, *F*(4,292) = 1.50, *p* = 0.20, $\eta_{p}^{2}$= 0.02, suggesting that the performance of both groups followed the same learning slope, though the sitting condition tended to produce greater overall word recall. For AVLT recall, a 2-Group x 2-Trial (short-delay, long-delay) repeated measures ANOVA showed a main effect of time, *F*(1,72) = 6.21, *p* = 0.02, $\eta_{p}^{2}$= 0.08, both groups remembered more words at the short delay than the long delay. Neither the main effect of group, *F*(1,72) = 1.32, *p* = 0.26, $\eta_{p}^{2}$= 0.02, or the Group x Trial interaction for recall were significant, *F*(1,72) = 0.29, *p* = 0.60, $\eta_{p}^{2}$= 0.004. The pattern of results remained nearly identical when VO_2_ peak was included as a covariate in the analyses. For the AVLT learning, the significant main effect of group, *F*(1,71) = 4.28, *p* = 0.04, $\eta_{p}^{2}$= 0.06, and time, *F*(4,284) = 7.96, *p*<0.001, $\eta_{p}^{2}$= 0.10, remained. Other effects remained non-significant.

**Table 2:** Cognitive outcomes for the sitting and treadmill walking groups.

	Sitting *n* = 38	Treadmill *n* = 37
AVLT: Total Learning	56.18 (7.73)	52.32 (7.60)*
AVLT: Short Delay Recall	12.32 (2.50)	11.76 (2.33)
AVLT: Long Delay Recall	11.95 (2.68)	11.30 (2.57)
AVLT: Trial 1 Recall	7.58 (1.35)	6.57 (1.68)
AVLT: Trial 2 Recall	10.65 (2.20)	9.46 (1.92)
AVLT: Trial 3 Recall	11.74 (1.84)	10.95 (2.01)
AVLT: Trial 4 Recall	12.84 (2.32)	12.46 (1.74)
AVLT: Trial 5 Recall	13.37 (1.75)	12.89 (1.85)
PASAT: Total Correct	138.47 (21.18)	126.64 (24.65)**
PASAT: Block 1 Correct	43.50 (5.92)	41.19 (6.81)
PASAT Block 2 Correct	37.58 (6.97)	33.14 (7.44)
PASAT Block 3 Correct	31.74 (6.63)	29.86 (7.79)
PASAT Block 4 Correct	25.66 (5.50)	22.46 (7.23)

Data include mean (SD).

NOTE: AVLT = Rey Auditory Verbal Learning Test.

PASAT = Paced Auditory Serial Attention.

For all AVLT variables, the number represents the number of words correctly recalled.

For all PASAT variables, the numbers represent the number correct.

*Indicates a significant main effect for group (*F*(1,73) = 4.75, *p* = 0.03, $\eta_{p}^{2}$= 0.06).

**Indicates a significant main effect for group (*F*(1,73) = 4.97, *p* = 0.03, $\eta_{p}^{2}$= 0.06).

There were main effects for Trial/Block in both the AVLT and PASAT (*p*<0.001).

Note: There was no significant Group x Trial interactions.

For the PASAT, total number of correct responses was lower in the treadmill walking group relative to the sitting group; the main effect was significant, *F*(1,73) = 4.97, *p* = 0.03, $\eta_{p}^{2}$= 0.06. A 2-Group x 4-Trial (Block) repeated measures ANOVA showed the expected main effect of trial, *F*(3,219) = 288.21, *p*<0.001, $\eta_{p}^{2}$= 0.80, with decreased correct responses for both groups with increased task speed. The Group x Trial interaction was not significant, *F*(3,219) = 1.56, *p* = 0.20, $\eta_{p}^{2}$= 0.02, suggesting that performance of both groups followed the same slope, though the sitting condition tended to produce more correct responses. The pattern of results was also similar when VO_2_ peak was included as a covariate in the analyses. The significant main effects of time, *F*(3,213) = 9.54, *p*<0.001, $\eta_{p}^{2}$= 0.12, and group, *F*(1,71) = 4.94, *p* = 0.03, $\eta_{p}^{2}$= 0.07, remained with all other effects remaining non-significant. Notably, standardized *z-*scores suggest that both AVLT and PASAT performance were within the average range for both groups, although some scores for the treadmill group on the PASAT approached a standard deviation below the normative mean (Table 3).

**Table 3:** Standardized *z*-scores relative to age-corrected norms for the cognitive outcome variables as a function of group.

	Sitting *n* = 38	Treadmill *n* = 37
AVLT: Total Learning	0.07 (1.17)	-0.48 (1.08)
AVLT: Short Delay Recall	0.36 (1.08)	0.13 (0.98)
AVLT: Long Delay Recall	0.24 (1.08)	-0.08 (1.13)
AVLT: Recognition	-0.08 (1.03)	0.01 (0.90)
AVLT: Trial 1 Recall	0.38 (0.78)	-0.20 (0.96)
AVLT: Trial 2 Recall	0.48 (1.11)	-0.08 (0.94)
AVLT: Trial 3 Recall	0.18 (0.98)	-0.28 (1.06)
AVLT: Trial 4 Recall	0.26 (1.24)	0.05 (0.94)
AVLT: Trial 5 Recall	0.35 (1.07)	0.00 (1.16)
PASAT: Total Correct	-0.41 (0.90)	-0.91 (1.05)
PASAT: Block 1 Correct	-0.35 (1.38)	-0.89 (1.58)
PASAT Block 2 Correct	-0.18 (0.89)	-0.75 (0.95)
PASAT Block 3 Correct	-0.55 (0.86)	-0.80 (1.01)
PASAT Block 4 Correct	-0.35 (0.82)	-0.83 (1.08)

Data include mean (SD).

NOTE: AVLT = Rey Auditory Verbal Learning Test

PASAT = Paced Auditory Serial Attention

Finally, there were significant between-group differences for all measures of typing speed (Table 4). Individuals in the treadmill-walking group typed significantly slower on indices of both gross and net WPM relative to the sitting controls (*p*<0.05). Individuals in the treadmill walking group also had significantly worse accuracy than those who sat during the task (*p*<0.05). Finally, ANCOVA results for typing speed when VO_2_ peak was added as a covariate remained significant for gross WPM, *F*(1,71) = 9.61, *p* = 0.003, net WPM, *F*(1,71) = 11.95, *p* = 0.001, and accuracy, *F*(1,71) = 7.75, *p* = 0.007.

**Table 4:** Typing speed outcome variables for sitting and treadmill walking participants.

	Sitting n = 38	Treadmill n = 37	t value	*p* value	Cohen’s d
Typing: Gross WPM	60.45 (16.61)	49.03 (15.34)	3.09	0.003	0.71
Typing: Net WPM	53.95 (17.02)	41.14 (15.40)	3.42	0.001	0.79
Typing: Accuracy (%)	0.88 (0.07)	0.82 (0.13)	2.53	0.014	0.58

Data include mean (SD) unless otherwise specified.

WPM = words per minute

Gross WPM—total words typed per minute

NET WPM = Gross words—incorrect words; adjusted speed per minute

Accuracy = percent of words typed correctly

## Discussion

The primary purpose of this study was to compare group differences on psychometrically-validated measures of attention, processing speed, learning and memory, and typing performance during a period of sitting or slow treadmill walking, such as what might be used on a treadmill desk in the workplace. Participants in the treadmill walking group exhibited significantly lower levels of learning on the AVLT, and worse overall performance on the PASAT (processing speed, attention, and working memory) and the typing test compared to the sitting group; however, the performance of both groups followed the same slope across trials (time). Relative to the sitting group, total learning on the AVLT was ~7% lower, total number of correct responses on the PASAT was ~9% lower, and net typing speed was ~13 WPM less in the treadmill walking group. Notably, however, recall on the AVLT was intact for both the short- and long-delay periods and performance of both groups was within the normal range for both the AVLT and PASAT relative to normative data, suggesting some modest between-groups differences but intact overall performance.

Many of the studies examining performance outcomes associated specifically with treadmill workstations have focused on typing or computer skills (e.g., speed of using a mouse). Relative to typing outcomes, the present study was in agreement with several other studies [27,30]. However, cognitive outcomes while simultaneously walking on a treadmill are much less reported. Thus, the measurement of validated cognitive outcomes was a significant strength of the present study. In addition, our measures included commonly used neuropsychological tasks that may be more sensitive to cognitive alterations and allow for comparisons against normative databases that are not provided in previous studies.

Consistent with John et al. [27], the present study revealed that treadmill walking tended to result in modestly diminished cognitive performance relative to sitting. However, our findings contrast John et al. [27] for attention and processing speed, who showed no difference during treadmill walking. Similarly, our findings are generally in contrast with Alderman et al. [29] and Ohlinger et al. [28] who reported that treadmill walking did not significantly influence cognitive tasks compared to sitting.

We highlight several possible explanations for the contrasting findings. First, the cognitive tasks performed differ among these studies. John et al. utilized the Stroop Color Word test (SCWT) (attention and processing speed) and GRE tests for mathematical reasoning and reading comprehension [27]; Alderman et al. utilized the Stroop test (attention and processing speed), a modified flanker test (attention), and a test of reading comprehension [29]; Ohlinger et al. also utilized the SCWT (attention and processing speed), but also the Auditory Consonant Trigram test (working memory and attention) [28]; whereas, the present study utilized the AVLT (learning and memory) and PASAT (processing speed, attention, and working memory) tests. Thus, future replication and use of tasks that examine more domains than memory and attention/executive tasks as well as mundane tasks like those used in some workplaces is needed.

Second, experimental designs and treadmill speeds were different. Our study was unique to past research in that the aforementioned studies were comprised of a within-subjects design where all subjects were exposed to a sitting and walking task, while our study was a randomized between-groups comparison. In addition, the present study used a treadmill speed of 1.5 mph; whereas, John et al. [27] and Ohlinger et al. [28] used a speed of 1.0 mph and Alderman et al. [29] used a self-selected speed ranging between 0.5–2.5 mph. Our findings are similar to Alderman et al. [29] and Ohlinger et al. [28] in that performance remained in the average range relative to the normative samples for both groups, despite significant differences in between-groups functioning.

As noted above, the majority of the research examining the effect of treadmill workstations have focused on typing [23], and other similar outcomes such as mouse-use ability [23,27] or transcription speed [24]. Our results for typing speed and efficiency is in agreement with others that typing efficiency is reduced while walking on a treadmill relative to sitting [26]. Indeed, it appears that walking on the treadmill more heavily affects typing speed performance than it does cognitive-type outcomes. Nevertheless, Tudor-Locke et al. recently noted the lack of research identifying the impact of practice on these and other outcomes [26]. It is possible that typing and fine-motor activities could be significantly improved with sustained practice on a treadmill, but existing studies (including the present study) have not yet examined this long-term. Perhaps an implication of the current study is that some jobs that require little motor skill (e.g., speaking on the phone or reading reports) could be completed on a treadmill, whereas other jobs that require extended typing or rapid processing speed should not use treadmill desks.

However, given that physical activity decreases various health risks [2–6], it is important to consider how the health benefits from using treadmill desks may outweigh the deficits in fine-motor skills and rapid processing speed. For example, obese individuals tend to sit more than lean individuals [51]. However, increased physical activity reduces the risk of obesity, even if that change in physical activity is small [52,53]. Simple daily activities outside of exercise, such as walking, fidgeting, and maintaining posture can be effective in reducing weight gain [51,54]. Using treadmill desks in the workplace capitalizes on these findings by providing employees the opportunity to increase their daily activity and physical health. In fact, during a one-year workplace intervention using treadmill desks, employees exhibited significant increased daily physical activity, decreased sedentariness, and modest weight loss, especially in those who were obese [19]. The use of treadmill desks has helped address health concerns by promoting simple physical activity in the workplace that fosters participation from employees, which may prove to be more beneficial long term than the decreases seen in productivity. Given this information, despite the between-groups differences in the current findings, the overall performance of both groups was generally within the average. Thus, it is likely that the benefit of using a treadmill desk outweighs the possible risks, particularly in obese individuals.

The current results of some decreases in function during exercise can be contrasted with a large body of literature showing improved cognitive performance following acute bouts of exercise [55,56]. The primary difference between our findings and those showing improvements after acute exercise is the timing of the exercise. Meta-analyses [31,32] showed decreased cognitive performance during exercise (primarily during the first 20-minutes of exercise), but improved cognitive performance following exercise. In-line with the results of the meta-analysis, treadmill walking during cognitive testing likely involves increased attention allocation that can decrease performance, whereas post-exercise cognitive improvements are associated with increased arousal and subsequent improvements in attention and cognitive skills [32], although, as outlined earlier, there is a large difference between speed-related outcome variables and accuracy-related outcomes depending on the intensity of the exercise [33].

This study has several strengths including: one of the largest sample sizes to date for treadmill workstation studies, a randomized design, and well-validated cognitive tests. The study also includes several limitations. One limitation of this study is that the tests were performed during only one session and did not examine potential learning/practice effects that could occur over time with consistent treadmill workstation use. In addition, the between-groups design and absence of a baseline testing session does not allow us to rule out the possibility that any findings between groups were present *a priori*. A second limitation is the lack of familiarity of the participants with the treadmill desk. It is possible that the positive findings in, for example, the AVLT were due to increased attention to the treadmill due to lack of familiarity with the treadmill setup. Future studies in experienced treadmill desk users are needed to address this possibility. Third, the sample consisted of university students that may not be representative of individuals in the day-to-day workforce. Fourth, the present study only examined one speed (1.5 mph) relative to sitting. It is possible that cognitive outcomes are influenced differently based on speed. The optimal treadmill speed for cognitive and performance outcomes is not currently known. Fifth, the height of the desk was not scalable, so the height of the keyboard for the typing task was not optimal for each participant. Finally, posture between sitting and standing is a potentially confounding variable that cannot be accounted for using the current study design.

In conclusion, increased physical activity and reducing sedentary time are important individual and public health objectives. We support the use of effective strategies to improve these health and functional outcomes. As noted in other studies, using a treadmill in the workplace or in other venues has the potential to increase physical activity and energy expenditure. However, the results of this study suggest that treadmill desks may result in modestly lower performance for some cognitive outcomes and/or fine-motor outcomes relative to sitting, although performance generally remains in the average range. Consideration of these potential effects is needed. Additional research is especially needed to determine if adaptation to a treadmill workstation occurs and to what extent this influences cognitive and work-related performance.

## Supporting Information

S1 Dataset Condition_number—0 = sitting; 1 = walking; BMI—Body Mass Index; AVLT—Rey Auditory Verbal Learning Test; PASAT—Paced Auditory Serial Addition Test. (CSV)
